# Cell-Penetration Peptide-Conjugated Octominin II Hybrid Inhibits the Multidrug-Resistant *Shigella flexneri*

**DOI:** 10.3390/antibiotics15101033

**Published:** 2026-10-16

**Authors:** Jayasinghage Nirmani Chathurnagika Jayasinghe, Chamilani Nikapitiya, Vijaya R. Dirisala, Tae-Won Kim, Mahanama De Zoysa, Ilson Whang

**Affiliations:** 1 College of Veterinary Medicine, Chungnam National University, Daejeon 34134, Republic of Korea; nirmanijay@o.cnu.ac.kr (J.N.C.J.); chamilani14@cnu.ac.kr (C.N.); taewonkim@cnu.ac.kr (T.-W.K.); 2 Department of Biotechnology, School of Biotechnology and Pharmaceutical Sciences, Vignan’s Foundation for Science, Technology and Research, Guntur 522213, Andhra Pradesh, India; dean_pcf@vignan.ac.in; 3 National Marine Biodiversity Institute of Korea (MABIK), 75, Seochun-gun 33662, Chungchungnam-do, Republic of Korea

**Keywords:** antibacterial, cell penetration, DNA damage, *Shigella flexneri*, TAT-Octominin II

## Abstract

**Background/Objectives:** The genus *Shigella* is one of the major pathological agents responsible for diarrhea and diarrhea-related mortality worldwide. It shows a decreasing sensitivity to most conventional antibiotics available today. **Methods:** Hence, an antimicrobial peptide, Octominin II, was conjugated to a cell-penetrating peptide (HIV-TAT) named TAT-Octominin II, and its antibacterial effect against *Shigella flexneri* was investigated. **Results:** TAT-Octominin II displayed minimum inhibitory and bactericidal concentrations of 10 and 25 µg/mL, respectively. It showed rapid membrane-permeabilizing activity, as observed in the ONPG assay. TAT-Octominin II treatment altered the cell membrane integrity and causing membrane damage in *S. flexneri.* Moreover, TAT-Octominin II increased oxidative stress, as evidenced by the upregulation of antioxidant genes, such as sodA, sodB, katG, oxyR, and soxR. Furthermore, TAT-Octominin II bound to genomic DNA and RNA, which likely inhibited the transcription and translation of bacterial proteins. In addition, TAT-Octominin II possessed lipopolysaccharide neutralization activity. In vitro toxicity analysis was performed using mammalian Raw 264.7 and HEK 293 cells, which showed no toxicity at lower concentrations (0–100 µg/mL). **Conclusions:** Overall, our results demonstrate that TAT-conjugated Octominin II exhibits enhanced antibacterial activity compared to Octominin II alone, suggesting that TAT-mediated delivery of AMPs could be a promising strategy to improve the therapeutic efficacy of AMPs.

## 1. Introduction

Shigella is a genus of bacteria belonging to the family Enterobacteriaceae. These highly infectious Gram-negative bacteria cause shigellosis, also known as bacillary dysentery. There are four known clinically important species of *Shigella*; *S. flexneri*, *S. dysenteriae*, *S. boydii*, and *S. sonnei*. Shigella is a major contributor to diarrheal diseases worldwide, causing approximately 125 million diarrheal cases annually [1]. It is also acknowledged as the main cause of mortality and morbidity due to diarrheal diseases in children aged <5 years [2]. Among *Shigella* spp., *S. flexneri* is the main etiological agent of diarrhea in developing countries [3]. Shigella has a very low infective dose and ingestion of approximately 10–100 organisms may be sufficient to cause infection and subsequent shigellosis [4]. *Shigella* spp. are resistant to most of the available antibiotics, creating obstacles to treating shigellosis in developing countries with restricted medical facilities. Antibiotics such as ampicillin, chloramphenicol, trimethoprim/sulfamethoxazole, and tetracycline are not prescribed as first-line drugs for treating shigellosis due to the rapid development of resistance. Moreover, growing resistance to cephalosporins has become a vital problem since it is an alternative drug for shigellosis [5]. *S. flexneri* has several drug-resistant mechanisms, including decreased membrane permeability, active efflux pumps, drug-target overexpression and mutation, and inactivation of drug-target enzymes [2]. Therefore, the World Health Organization (WHO) has listed *Shigella* spp. as one of the major threats by including it on its priority pathogen list that requires urgent attention to developing new antibiotic agents [6]. Understanding the potentially pressing issue of Shigella, scientists are exploring different approaches to eliminate the burden of Shigella infections.

Antimicrobial peptides (AMPs) are a growing area of research in the field of antimicrobial resistance. AMPs can be found in all-natural sources, and synthetic AMPs have also been applied to control drug-resistant microorganisms. AMPs exert inhibitory activity through different mechanisms. They can either directly kill microorganisms by damaging the cell membrane or disrupting membrane-bound activities. AMPs exert inhibitory activity through different mechanisms. They can either directly kill microorganisms by damaging the cell membrane or disrupting membrane-bound activities. AMPs can also exert indirect antimicrobial effects by modulating innate immune responses [7]. Several approaches have been explored to boost the activity of AMPs, including modifying the peptide sequence to enhance its superior physicochemical properties, such as net charge, hydrophobicity, and amphipathicity [8]. Another strategy is to combine AMPs with other molecules, such as nanoparticles, liposomes, and cell-penetrating peptides (CPPs), which can improve their delivery and stability, allowing them to reach the target site effectively [9,10]. CPPs are small peptides with 10–40 amino acids. They deliver bioactive therapeutic cargo to cells without causing major damage to the cell membrane using different mechanisms, mainly endocytosis [11]. There are approximately 1700 CPPs documented in CPPsite 2.0 [12]. Together, AMPs and CPPs are categorized into the membrane-active peptide family [13]. CPPs such as human immunodeficiency virus type I trans-activator of transcription (HIV-1 TAT), penetratin (pAntp), polyarginines, DPV1047, MPG, and Pep-1 are a few examples of CPPs that have been studied extensively [14]. Among them, the HIV-1 TAT peptide has been studied as an efficient carrier for treating various human diseases in conjugation with certain drugs, small molecules, liposomes, nanoparticles, nucleotides, and peptides [15].

AMP activity can vary among species in terms of mode of action, dose, and sensitivity. Our research group previously characterized Octominin II, an 18-amino-acid antimicrobial peptide derived from the previously developed Octominin peptide. Octominin II is a cationic peptide with a net positive charge and moderate hydrophobicity and has previously demonstrated potent antifungal activity against *Candida* spp. through multiple mechanisms, including disruption of membrane integrity, induction of oxidative stress, interaction with nucleic acids, and inhibition of biofilm formation [16]. These previously reported antimicrobial properties suggested that Octominin II could serve as a promising scaffold for further modification. However, Octominin II alone requires relatively high concentrations to inhibit certain bacteria, including *S. flexneri*. Therefore, in the present study, Octominin II was conjugated with the cell-penetrating peptide TAT (YGRKKRRQRRR) to enhance its uptake and antibacterial activity. *S. flexneri* was used as a model organism to evaluate the activity of TAT-Octominin II. Functional properties of the newly modified peptide was analyzed by investigating its modes of action against *S. flexneri*, including membrane-associated effects, DNA- and RNA-binding activity, lipopolysaccharide (LPS)-neutralizing activity, and toxicity.

## 2. Results

### 2.1. Multidrug Resistance of S. flexneri

Species verification of the bacterium was confirmed by 16S rRNA gene sequencing and a motility test (Supplementary Figure S1). As *S. flexneri* is non-motile compared to *E. coli*, the motility test results confirmed that the selected bacterium belonged to *Shigella* spp., and the 16S rRNA sequencing results confirmed that the isolated *Shigella* culture was *S. flexneri* (EzBioCloud; 99.9% sequence identity). To screen the antibiotic resistance level of *S. flexneri*, it was tested with 19 antibiotics using the antibiotic disc diffusion assay. Among the antibiotics tested (Table 1), *S. flexneri* was susceptible to only three antibiotics: gentamicin, cefotaxime, and imipenem (15.7%). Intermediate susceptibility was observed for two antibiotics, ciprofloxacin and ofloxacin (10.5%), with resistance to the remaining 14 antibiotics (73.68%). This proves that the *S. flexneri* strain chosen for this study was a multidrug resistant bacterium (Table 1).

### 2.2. Design and Physicochemical Properties of TAT-Octominin II

**Figure 1:**
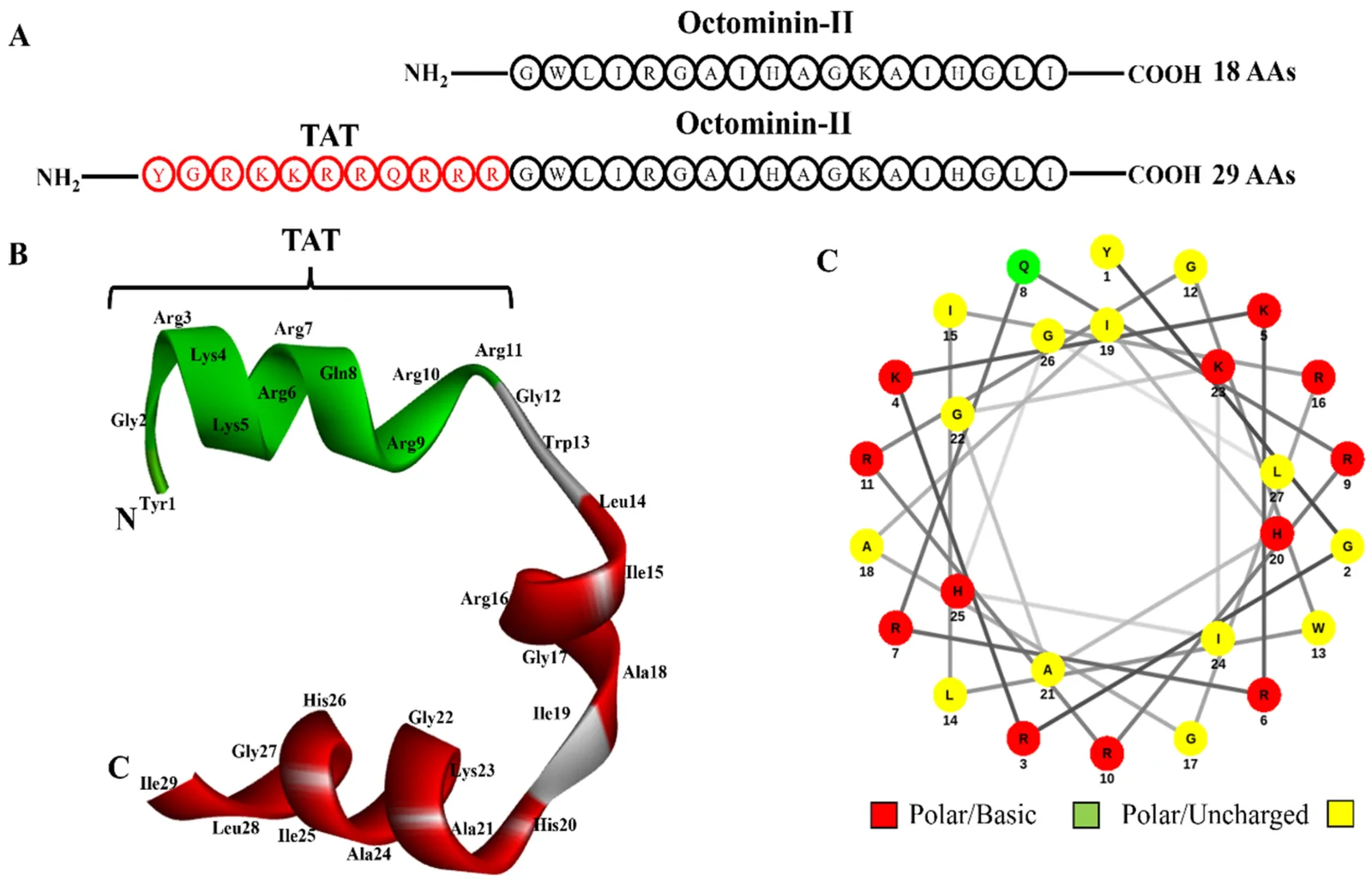
Amino acid sequence, predicted secondary structure, and the helical wheel projection of TAT-Octominin II. (**A**) Amino acid sequences of Octominin II and TAT-Octominin II. (**B**) Predicted secondary structure. (**C**) Helical wheel projection of TAT-Octominin II. Polar (basic), polar (uncharged), and nonpolar amino acids are indicated in red, green, and yellow, respectively.

TAT-Octominin II was designed to increase the antimicrobial activity and cell membrane permeability of Octominin II. The amino acid sequences of Octominin II and TAT-Octominin II are shown in Figure 1A. The predicted secondary structure of TAT-Octominin II showed α-helical and random coil structures (Figure 1B). The observed secondary structure may partly reflect the influence of TAT conjugation; however, the specific contribution of the TAT sequence to peptide helicity requires further comparative structural analysis. The helical wheel projection shows the placement of polar and nonpolar amino acids (Figure 1C). The molecular weight of TAT-Octominin II was 3425.10 Da, with a higher net positive charge (+10.00) than Octominin II (+2.46). The hydrophobicity of the peptide was 0.33, and the Boman index was 1.23 kcal/mol (Table 2).

### 2.3. MIC and MBC of TAT-Octominin II Against S. flexneri

**Figure 2:**
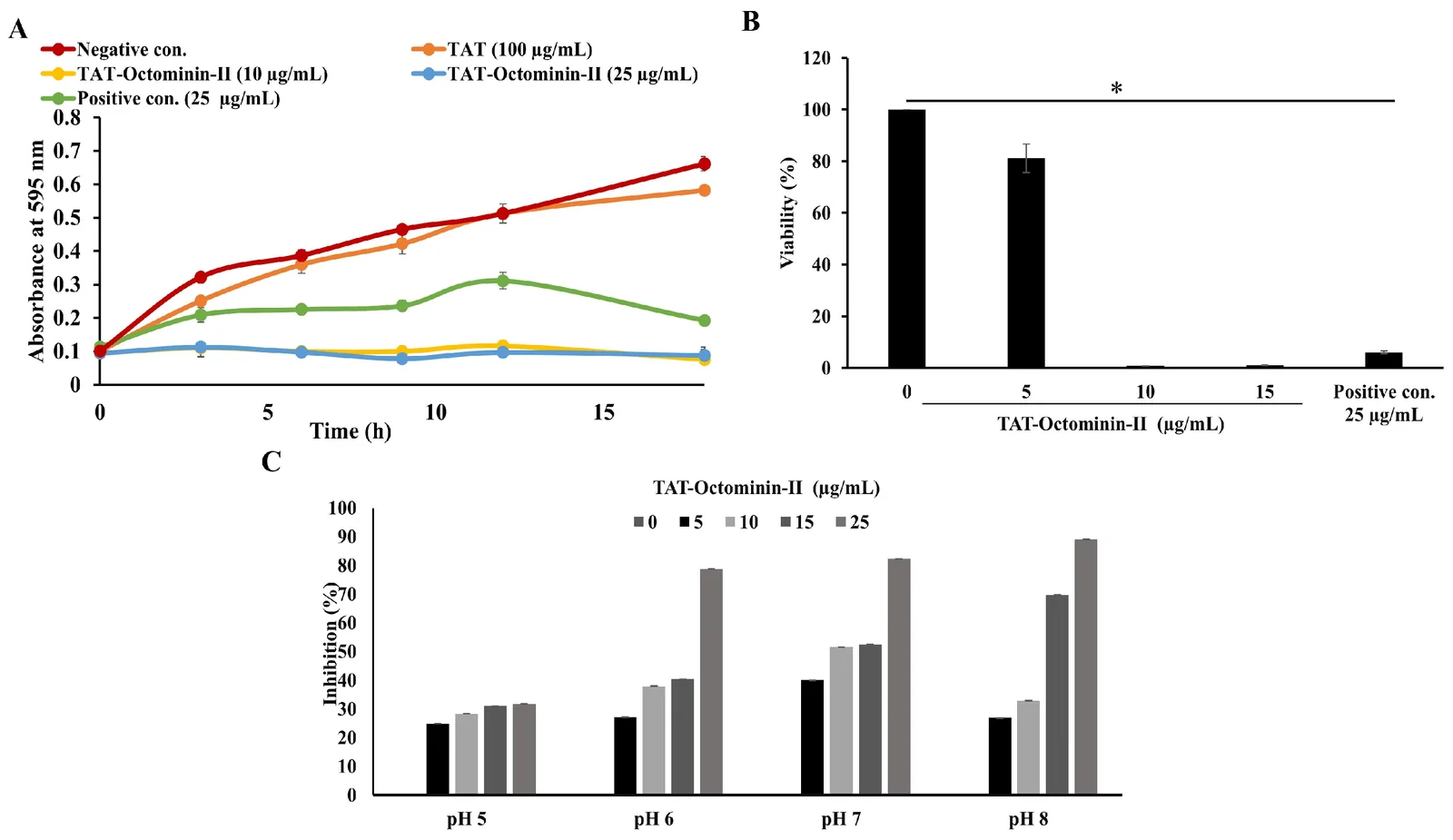
Time-kill kinetics, cell viability, and pH-dependent antibacterial activity of TAT-Octominin II against *S. flexneri*. (**A**) Time-kill kinetics of TAT-Octominin II against *S. flexneri*. Growth of *S. flexneri* was assessed after TAT-Octominin II treatment (0, 10, and 25 µg/mL) at 3 h intervals by measuring the optical density at 595 nm wavelength. Gentamycin (25 µg/mL) was used as the positive control. (**B**) Cell viability of *S. flexneri* after treatment with TAT-Octominin II was assessed via MTT assay. (**C**) pH dependent activity of TAT-Octominin II where inhibitory activity was assessed in a pH range of 5–8. * *p* < 0.05.

Octominin II and TAT-Octominin II peptides were tested against *S. flexneri* to determine the MIC and MBC. For Octominin II, the MIC and MBC values were 160 and 250 µg/mL, respectively, whereas TAT-Octominin II showed lower MIC and MBC values of 10 and 25 µg/mL, respectively. After conjugating the cell-penetrating TAT peptide, the initial screening proved that TAT-Octominin II had higher antimicrobial activity than Octominin II. Time kill-kinetics assay showed the time-dependent inhibition of *S. flexneri* by TAT-Octominin II, where MIC (10 µg/mL) and MBC (25 µg/mL) inhibited the growth of *S. flexneri* (Figure 2A). Moreover, only TAT (100 µg/mL)-treated samples were used to confirm whether it showed any antimicrobial effects, and the results indicated that TAT itself exerted no inhibitory activity against *S. flexneri*. The remaining viable biomass after TAT-Octominin II treatment was observed at MIC and MBC, as determined by the MTT assay (Figure 2B). The viability of *S. flexneri* was low at 10 (1.04%) and 25 µg/mL (0.83%) concentrations of TAT-Octominin II, compared to 5 µg/mL (81.13%), indicating good inhibition. The pH-dependent antibacterial activity of TAT-Octominin II was determined over a pH range (3, 4, 5, 6, 7, and 8). *S. flexneri* was not grown at acidic conditions, at pH 3 and 4. However, neutral and basic pH media boosted the growth of *S. flexneri*. The growth inhibition by TAT-Octominin II was calculated relative to that of the negative control. The inhibitory activity increased at pH 6, 7, and 8. The lowest activity was observed at pH 5 compared to the other tested pH values. Optimum growth of *S. flexneri* was observed at pH 7, and the optimum activity of TAT-Octominin II was also observed at pH 7 (82.33% at 25 µg/mL). Although the relative inhibition was higher at pH 8 (89.12% at 25 µg/mL), the growth of *S. flexneri* in the negative control was lower than that at pH 7. Therefore, all the remaining experiments were performed at pH 7.

### 2.4. TAT-Octominin II Changed the Cell Membrane Ultrastructure in S. flexneri

**Figure 3:**
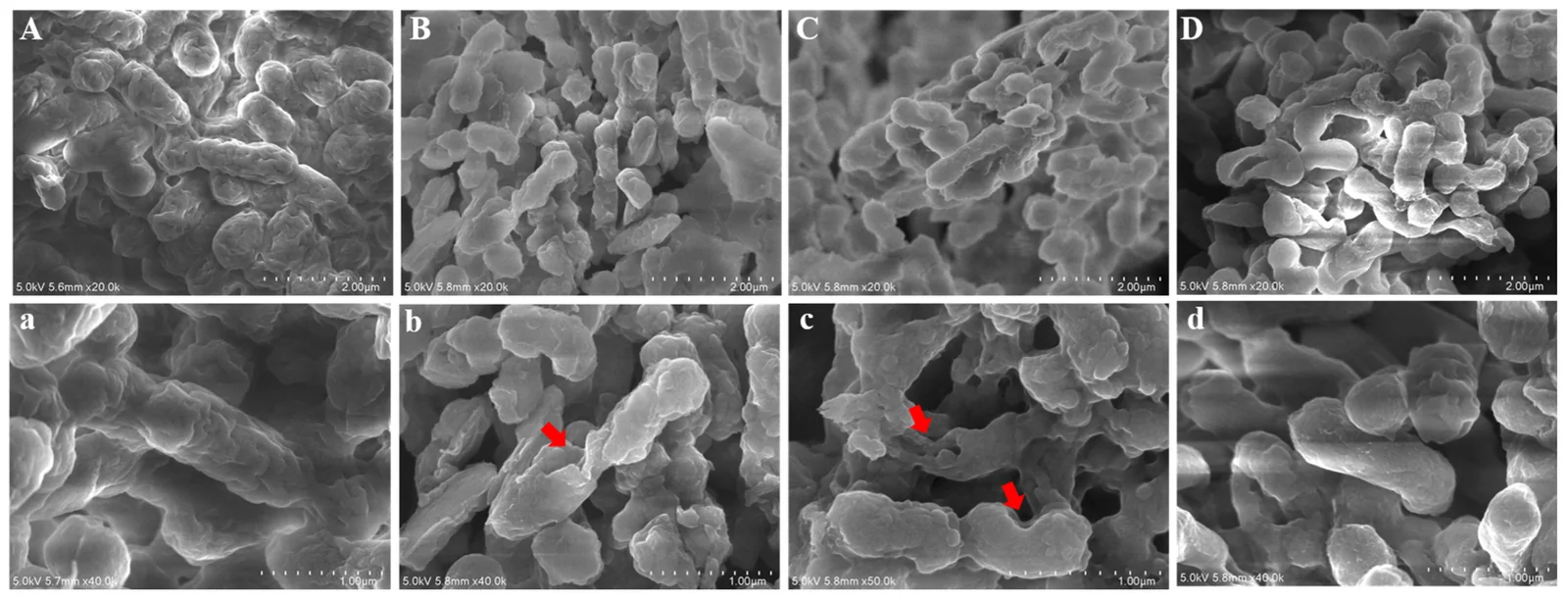
Effect of TAT-Octominin II on morphological and structural changes in *S. flexneri* examined by FE-SEM. (**A**–**D**) represent lower-magnification images (200 µm scale bars), whereas a-d represent the corresponding higher-magnification (1 µm scale bars) images. (**A**,**a**), untreated control group; (**B**,**b**), TAT-Octominin II at MIC (10 µg/mL); (**C**,**c**), TAT-Octominin II at MBC (25 µg/mL); and (**D**,**d**), gentamicin-treated positive control (25 µg/mL). Arrows indicate ruptured and morphologically defective cells.

*S. flexneri* was treated with TAT-Octominin II and observed using field emission microscopy (FE-SEM) to investigate the effects of TAT-Octominin II on *S. flexneri* cell morphology. The untreated negative control showed rod-shaped and non-impaired *S. flexneri* cells (Figure 3A). TAT-Octominin II treatment at the MIC (10 µg/mL) drastically altered the cell morphology, with ruptured and mutilated cells compared with the untreated negative control (Figure 3B,b). In addition, the MBC-treated (25 µg/mL) sample contained more lysed *S. flexneri* cells with clumps of dead cells and cell debris than the untreated sample (Figure 3C,c). The positive control, gentamicin-treated samples, also showed smooth cell surfaces and few ruptured cells (Figure 3D,d). SEM images confirmed that TAT-Octominin II severely altered the cell morphology of *S. flexneri*.

### 2.5. Increased Inner Membrane Permeability and Nucleic Acid Leakage in TAT-Octominin II Treated S. flexneri

**Figure 4:**
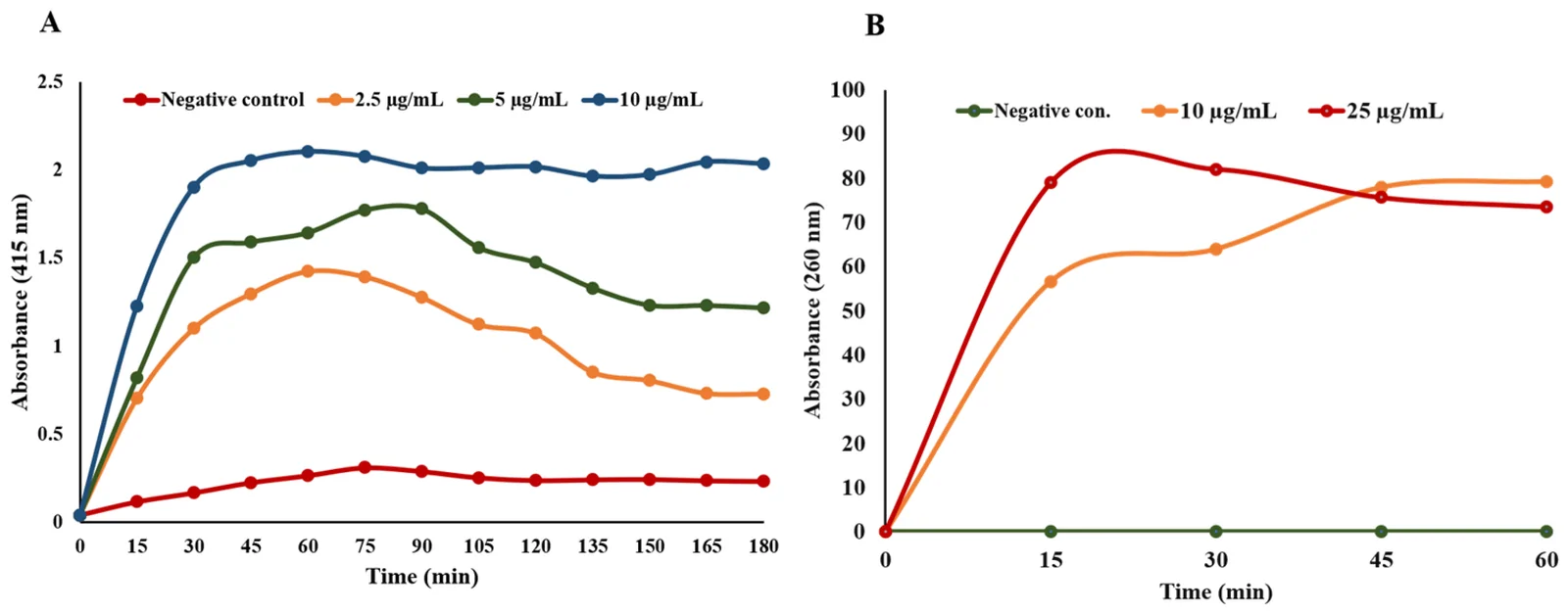
Effect of TAT-Octominin II on inner cell membrane permeability and nucleic acid leakage of *S. flexneri.* (**A**) The inner cell membrane permeability assay. Membrane permeability of *S. flexneri* was determined by measuring the release of β-galactosidase activity into the medium with ONPG as a substrate after treatment with the TAT-Octominin II (0, 2.5, 5, and 10 µg/mL) and negative control. (**B**) Nucleic acid leakage assay. Released nucleic acids were measured at 260 nm after treatment with TAT-Octominin II at different time intervals (MIC and MBC).

The o-Nitrophenyl-β-D-galactopyranoside (ONPG) assay was performed to determine the inner membrane permeability of the TAT-Octominin II treatment. ONPG is a colorless compound, but it develops a yellow color when hydrolyzed by the β-galactosidase enzyme to O-nitrophenol. β-galactosidase is an intracellular enzyme of bacteria, which leaks outside when the cell permeability is increased. TAT-Octominin II treatment increased the development of O-nitrophenol in a concentration-dependent manner (Figure 4A). After 30 min of TAT-Octominin II MIC (10 µg/mL) treatment, the cells displayed the highest absorbance, with an increasing trend observed in all treatments. TAT-Octominin II at 2.5 and 5 µg/mL reached a maximum level at 60 and 90 min, respectively, and showed a downward trend, while 10 µg/mL showed higher absorbance and remained at a plateau. These observations indicate that TAT-Octominin II increases the inner cell permeability of *S. flexneri*. The *S. flexneri* cultures used for the ONPG assay were plated at 1 h intervals to investigate the viability of the treated *S. flexneri*. Despite the observed high cell permeability at 2.5 and 5 µg/mL, *S. flexneri* remained viable (Supplementary Figure S2). However, the MIC (10 µg/mL)-treated sample showed a decreased *S. flexneri* viability. The results indicate that TAT-Octominin II changes cell membrane permeability, even at lower concentrations. However, the increase in cell membrane permeability alone does not account for the complete inhibition of *S. flexneri* (Supplementary Figure S2). The absorbance of the supernatants of the TAT-Octominin II-treated *S. flexneri* cultures was measured at 260 nm to measure nucleic acid (DNA and RNA) leakage from the cytoplasm (Figure 4B). TAT-Octominin II MBC (25 µg/mL)-treated samples showed an immediate spike of nucleic acid leakage, followed by a gradual decline, whereas MIC (10 µg/mL) showed a gradual release of nucleic acids.

### 2.6. TAT-Octominin II Changed Membrane Integrity and Permeability in S. flexneri Cells

**Figure 5:**
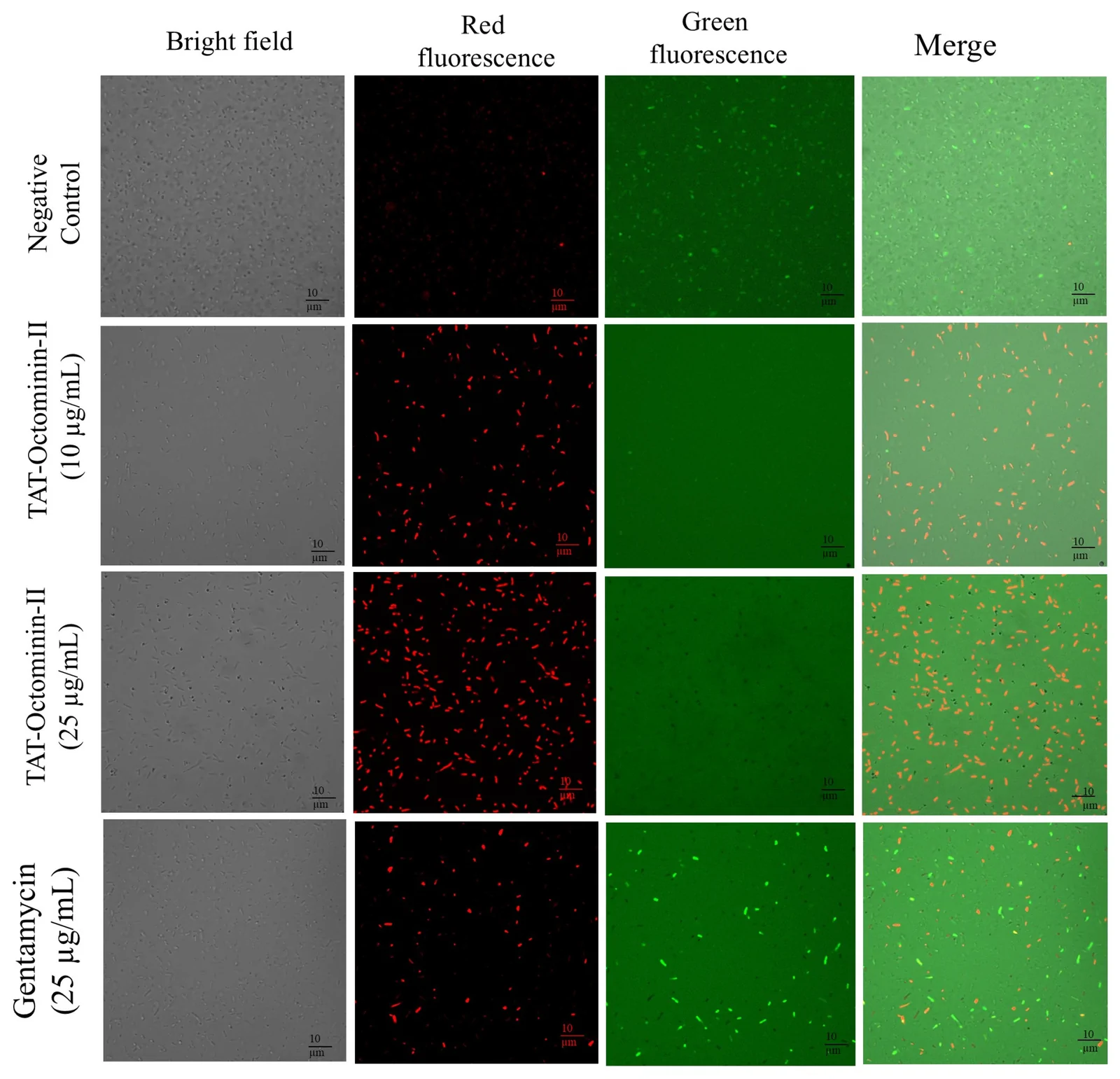
Effect of TAT-Octominin II on membrane integrity and permeability of *S. flexneri*. Untreated, TAT-Octominin II treated (MIC; 10 and MBC; 25 µg/mL), and Gentamycin (25 µg/mL) treated *S. flexneri* was incubated for 12 h at 37 °C. Then the *S. flexneri* cells were collected and stained with PI and FDA. Cells were visualized using a confocal laser microscope (CLSM). Red fluorescence (PI: dead cells) and green fluorescence (FDA: live cells) were observed at excitation wavelengths of 535 and 617 nm, respectively, and emission wavelengths of 488 and 535 nm, respectively.

The Propidium Iodide (PI)/Fluorescein Diacetate (FDA) assay provides insight into membrane damage and/or changes in cell membrane integrity, as PI cannot pass through viable cells. After treating *S. flexneri* with TAT-Octominin II (MIC: 10 µg/mL and MBC: 25 µg/mL), *S. flexneri* cells were stained with PI and FDA and observed via confocal laser microscopy (Figure 5). Untreated control samples exhibited higher green and lower red fluorescence signals, indicating more intact cells. TAT-Octominin II-treated cells exhibited stronger red fluorescence than the untreated control, indicating possible damage or altered cell membrane integrity in *S. flexneri*. The positive control (gentamicin 25 µg/mL) treated *S. flexneri* cells showed less red fluorescence than the TAT-Octominin II-treated samples.

### 2.7. TAT-Octominin II-Induced ROS in S. flexneri

**Figure 6:**
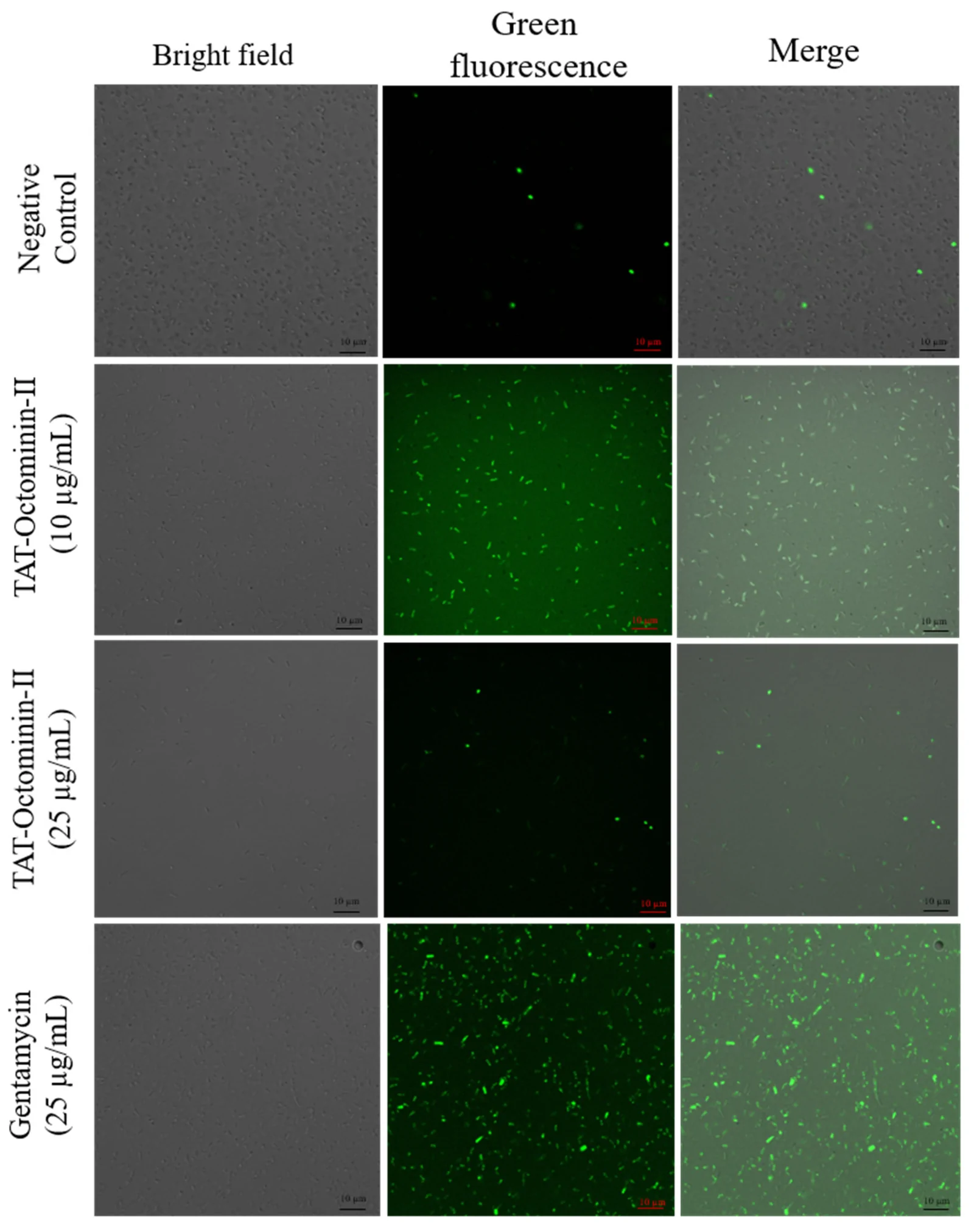
Effect of TAT-Octominin II on ROS production in *S. flexneri.* TAT-Octominin II (MIC; 10 and, MBC; 25 µg/mL) and gentamicin (25 µg/mL) treated *S. flexneri* and incubated at 37 °C for 10 h. Then, the cells were collected and stained with H_2_DCFDA) and observed using CLSM with green fluorescence at the excitation and emission wavelengths of 488 nm and 535 nm, respectively.

Reactive oxygen species (ROS) can be generated in response to antibiotics, and excessive ROS-associated oxidative stress can lead to bacterial cell death. *S. flexneri* was treated with TAT-Octominin II (MIC: 10 µg/mL and MBC: 25 µg/mL) and stained with H_2_DCFDA to monitor the generated ROS (green fluorescence). The untreated control emitted negligible green fluorescence, indicating low ROS production (Figure 6). The highest level of green fluorescence was observed in TAT-Octominin II (MIC) and the positive control (gentamicin 25 µg/mL)-treated *S. flexneri*. Because the MBC-treated sample had a low number of cells, it did not exhibit higher green fluorescence, as expected. These results demonstrate that TAT-Octominin II induces oxidative stress, which can lead to bacterial cell death. 

### 2.8. TAT-Octominin II Treatment Induced the Antioxidant Genes of S. flexneri

**Figure 7:**
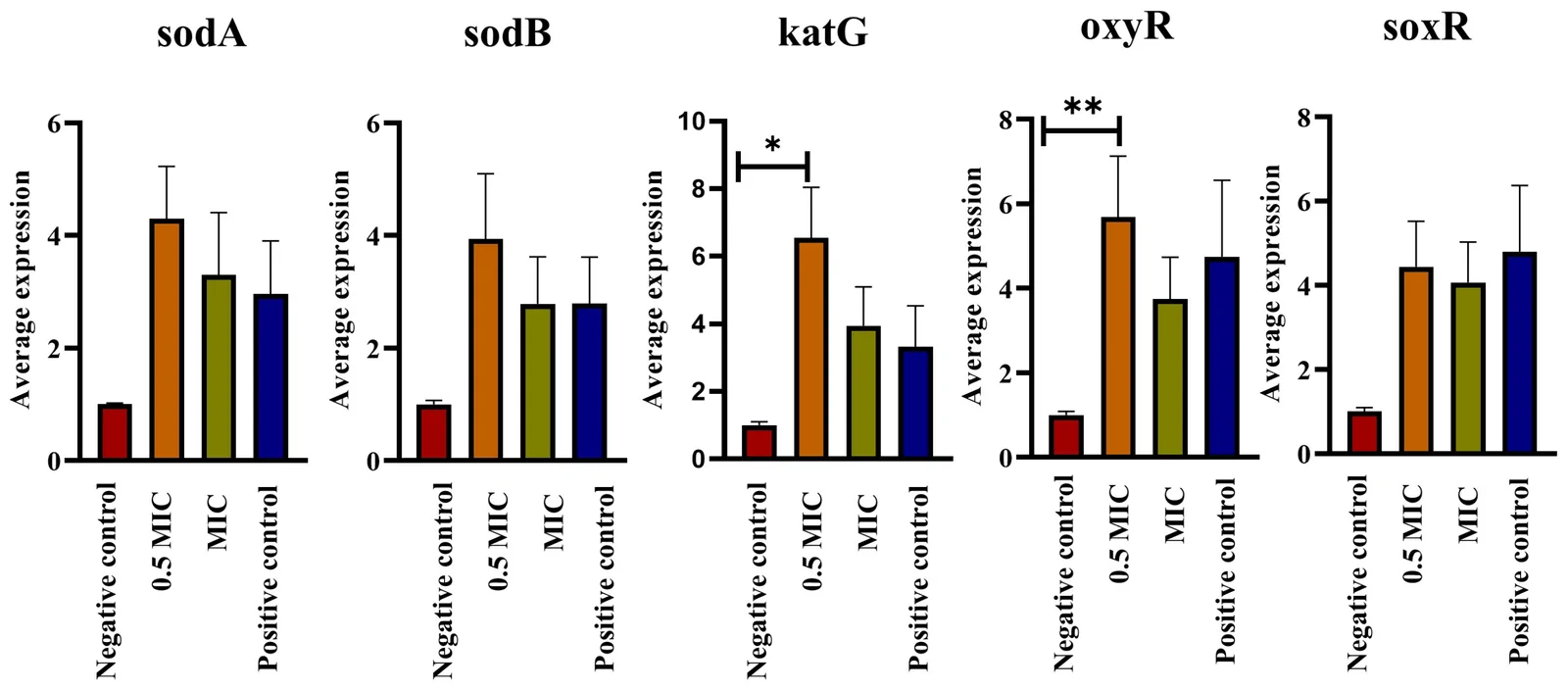
Expression of antioxidant genes in TAT-Octominin II treated *S. flexneri*. Antioxidant genes sodA, sodB, katG, oxyR, and soxR were evaluated after 3 h post-treatment of TAT-Octominin II. The relative mRNA expression fold values were calculated by following the 2^−(ΔΔCT)^ method (*n* = 3, * *p* < 0.05, ** *p* < 0.01). PC = Positive control (Gentamycin 25 µg/mL).

Transcriptional analysis was performed to analyze the antioxidant gene expression pattern of *S. flexneri* upon TAT-Octominin II treatment. All the tested antioxidant genes were upregulated, including oxyR (0.5 MIC = 5.69-fold, MIC = 3.74-fold), soxR (0.5 MIC = 4.44-fold, MIC = 4.07-fold), sodA (0.5 MIC = 3.94-fold, MIC = 2.79-fold), sodB (0.5 MIC = 4.29-fold, MIC = 3.30-fold), and katG (0.5 MIC = 6.54-fold, MIC = 3.94-fold), indicating increased oxidative stress in *S. flexneri* cells (Figure 7). All genes were upregulated by more than 2.5 times compared to the negative control at both TAT-Octominin II concentrations.

### 2.9. TAT-Octominin II Binds to Genomic DNA and RNA

**Figure 8:**
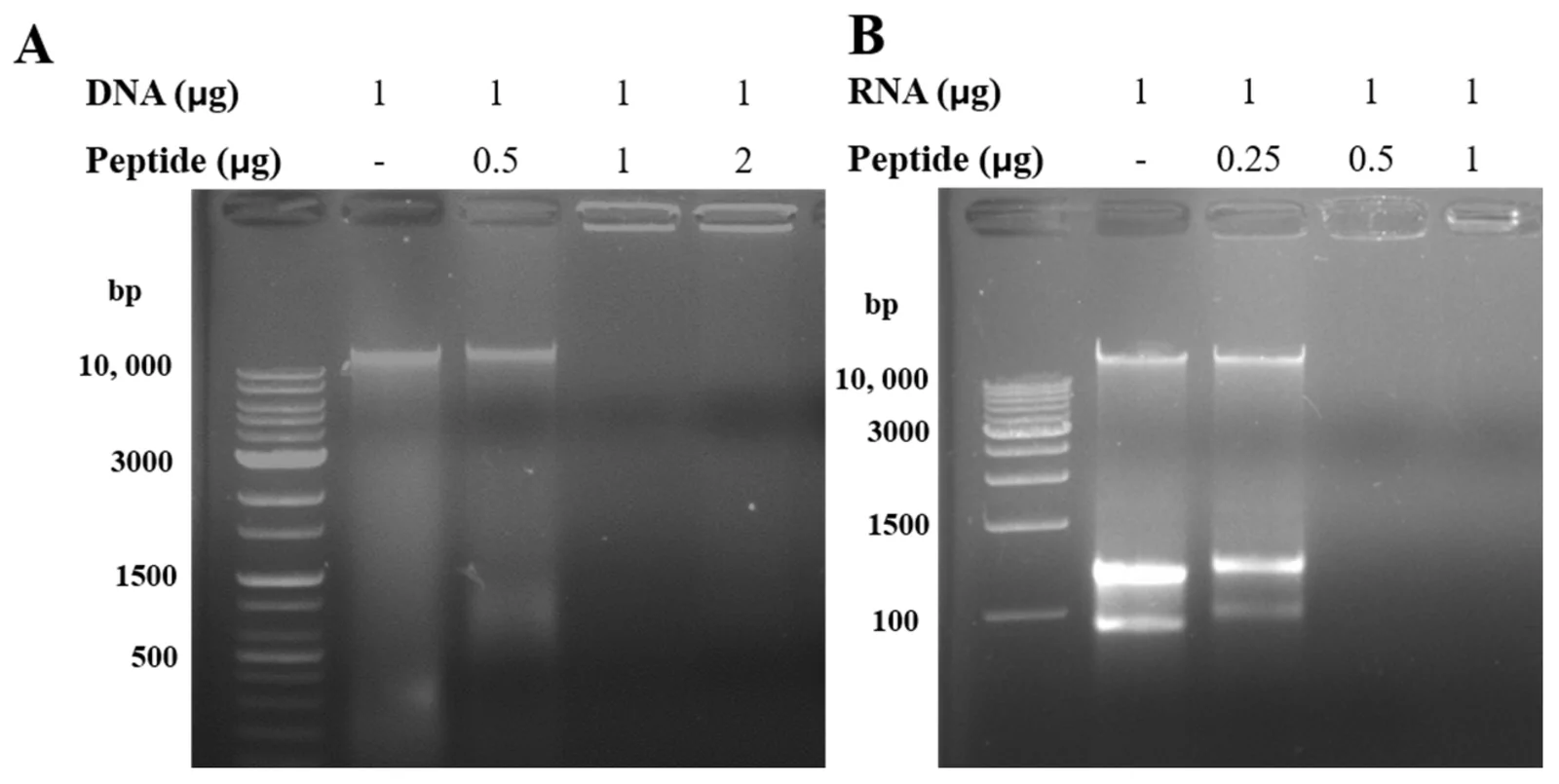
DNA and RNA binding activity of TAT-Octominin II. Genomic DNA and total RNA were isolated from *S. flexneri* and reacted with TAT-Octominin II at in vitro conditions at room temperature for 30 min and visualized via gel electrophoresis (1% Agarose gel). Binding pattern of (**A**) genomic DNA and (**B**) total RNA with TAT-Octominin II.2.10. LPS Neutralization Activity of TAT-Octominin II.

Because TAT-Octominin II displayed strong cell permeability activity, its effect on cellular macromolecular targets was assessed via DNA and RNA-binding assays using genomic DNA and total RNA isolated by *S. flexneri*. There we could perceive that TAT-Octominin II had strong genomic DNA binding activity at a ratio of DNA: TAT-Octominin II = 1:1 and 1:2 (*w*/*w*) as complete retardation of DNA migration was observed (Figure 8A). Moreover, it showed stronger total RNA-binding activity, where the RNA: TAT-Octominin II = 1:0.5 (*w*/*w*) reaction mixture showed complete RNA migration stagnation (Figure 8B).

### 2.10. LPS Neutralization Activity of TAT-Octominin II

**Figure 9:**
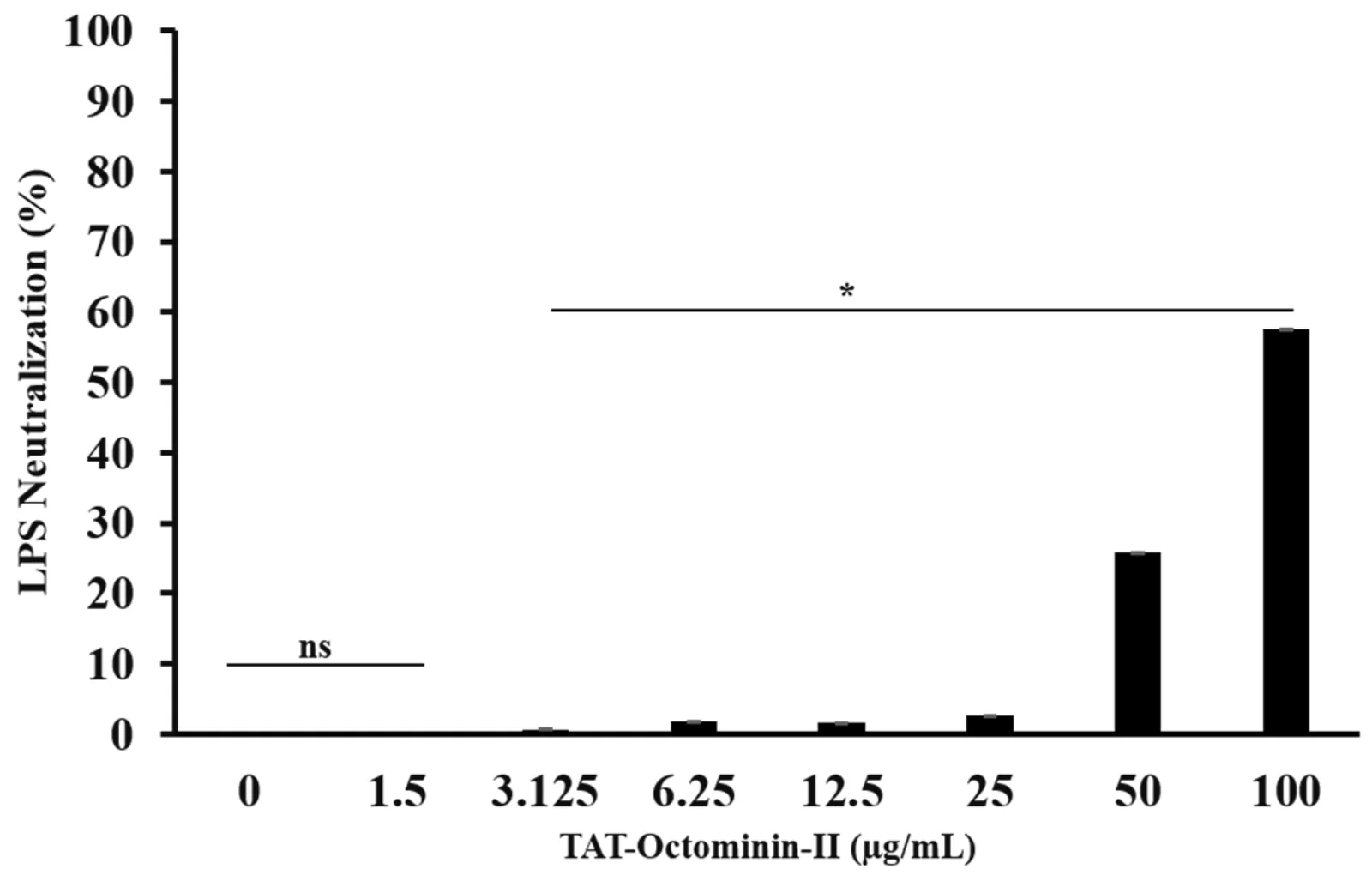
LPS neutralization activity of TAT-Octominin II. LPS (0.5 EU/mL) was treated with TAT-Octominin II (0–100 µg/mL) and incubated at 37 °C for 30 min where the remaining LPS after neutralization was measured using the LAL chromogenic endotoxin quantitation kit. The color change was measured at 410 nm and the calculations were done compared to the negative control with no TAT-Octominin II treatment. The error bars indicate the mean ± standard deviation (*n* = 3). ns = not significant. * *p* < 0.05.

Lipopolysaccharide (LPS) is the major component of the outer cell membrane of Gram-negative bacteria, that contributes to its pathogenicity. The ability of TAT-Octominin II to neutralize LPS was determined using the Limulus Amebocyte Lysate (LAL) chromogenic assay. It was observed that LPS was neutralized by TAT-Octominin II in a concentration-dependent manner, where low concentrations did not show considerable LPS-neutralizing activity, and a gradual increase in LPS-neutralizing activity was observed (from 25 µg/mL) when the concentration was increased (Figure 9).

### 2.11. Toxicity and Hemolysis Activity of TAT-Octominin II

**Figure 10:**
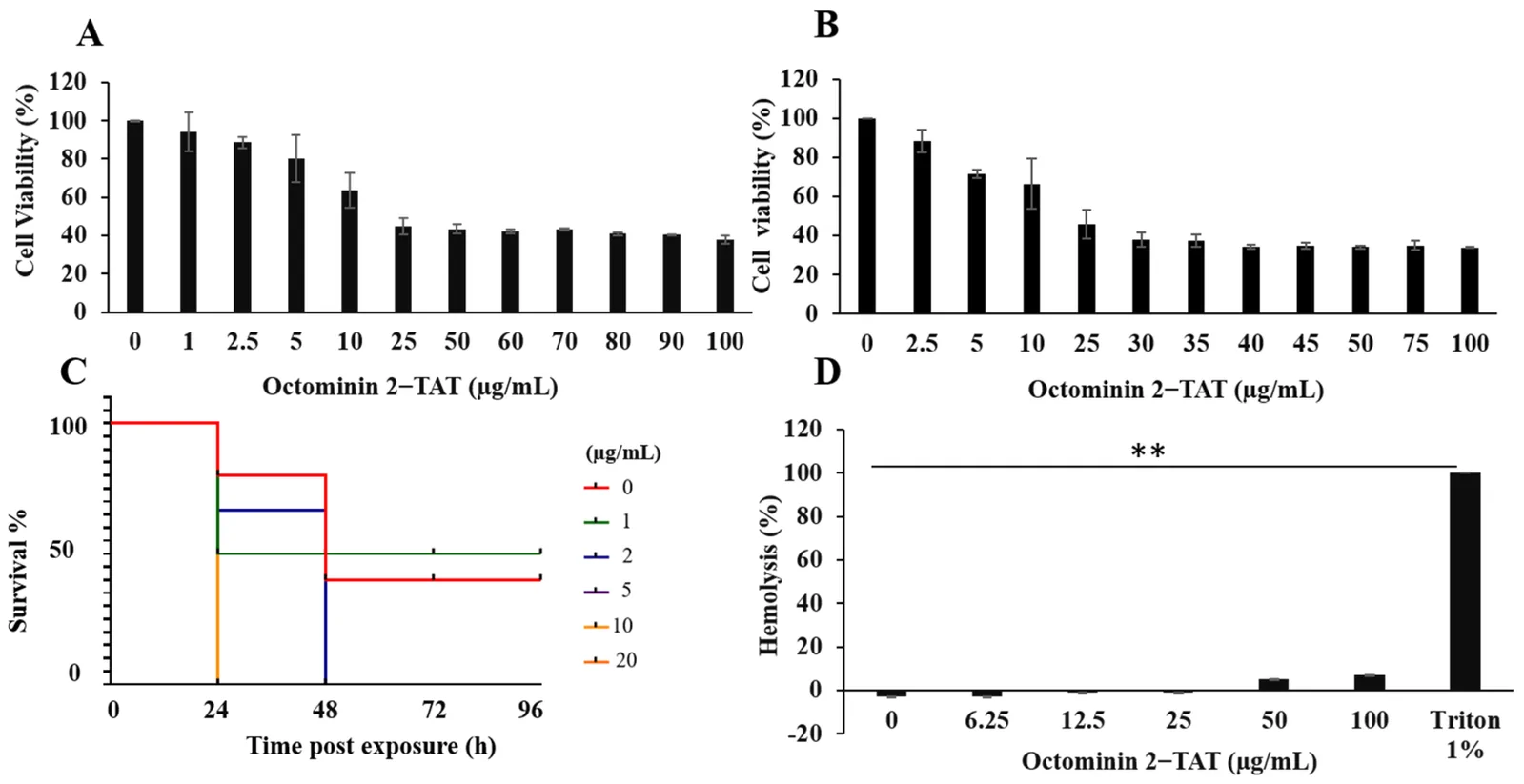
In vitro and in vivo toxicity profile of TAT-Octominin II. (**A**) TAT-Octominin II toxicity assessed with Raw 264.7 cells (0–100 μg/mL, *n* = 3). (**B**) TAT-Octominin II toxicity assessed with HEK-293 (0–100 μg/mL, *n* = 3). (**C**) In vivo toxicity assay of TAT-Octominin II assessed with zebrafish larvae (0–20 μg/mL, *n* = 10) where the zebrafish larvae were exposed to TAT-Octominin II and observed survival for 96 h. (**D**) Hemolysis activity of TAT-Octominin II assessed with murine red blood cells (0–100 μg/mL, *n* = 3). ** *p* < 0.01.

In vitro cytotoxicity was assessed using murine macrophage (Raw 264.7) cells (Figure 10A) and human embryonic kidney (HEK-293) cells (Figure 10B) treated with TAT-Octominin II (0–100 µg/mL). TAT-Octominin II exhibited toxicity at higher concentrations in both cell lines, with IC_50_ values of 7.86 and 8.75 µg/mL for Raw 264.7 and HEK 293 cells, respectively. In vivo toxicity analysis (Figure 10C) revealed that TAT-Octominin II exerted a high level of toxicity in zebrafish larvae (0–20 µg/mL), where >50% mortality was observed within 48 h at 5, 10, and 20 µg/mL. The calculated LD_50_ was 1.86 µg/mL. The hemolytic activity of TAT-Octominin II was tested using murine red blood cells (RBCs) (0–100 µg/mL). TAT-Octominin II showed low hemolysis activity compared to the positive control;Triton X-100 (10 µg/mL: Sigma Aldrich, Munich, Germany), with a hemolysis percentage of 7.07% even at 100 µg/mL (Figure 10D).

## 3. Discussion

Several drugs are being prescribed against *Shigella* infections, including ciprofloxacin as 1st line, mecillinam/ceftriaxone as second-line drug for children, and azithromycin as second- line drug for adults [17]. According to the 2015 National Antimicrobial Resistance Monitoring System (NARMS) report by the Centers for Disease Control and Prevention CDC-USA, *Shigella* was mostly resistant to streptomycin, followed by azithromycin, trimethoprim/sulfamethoxazole, and tetracycline [17]. Sethuvel et al. (2017) summarized the *Shigella* spp. resistance patterns in several regions [18]. According to that report, an increased pattern of resistance to ceftriaxone was identified in a multicenter study conducted in selected Asian countries (Korea, Taiwan, Singapore, Thailand, Vietnam, the Philippines, Hong Kong, and Sri Lanka). A study conducted in China revealed that *S. flexneri* was mostly resistant to ampicillin among the tested isolates (98.5%), followed by ticarcillin (95.0%) and trimethoprim/sulfamethoxazole (82.5%). However, the resistance patterns of *S. flexneri* and *S. sonnei* differ [19]. The *S. flexneri* strain used in this study showed intermediate susceptibility to ciprofloxacin and was resistant to 14 other antibiotics, a high level of antimicrobial resistance. Therefore, the *S. flexneri* strain used in this study represents a relevant model for providing insights into the current characteristics of virulence and antimicrobial resistance in *S. flexneri*.

Conjugating AMPs with CPPs to increase AMPs uptake is one approach used to increase the antimicrobial activity of AMPs. CPPs can transport cargo by interacting with cellular membranes. As CPPs contain more positively charged AAs, they generally exhibit cationic characteristics. The conjugation of TAT to Octominin II increased the overall net positive charge, which may enhance the electrostatic interactions with negatively charged structures, such as cell membranes. However, the net positive charge is sufficiently high to cause cell membrane damage, which can lead to unnecessary cytotoxicity exerted by the peptide. Most AMPs with +4 to +6 net charge show better antimicrobial activity and are considered to represent an optimum net charge of the peptide [20]. TAT-Octominin II exhibited MIC and MBC values 10 and 25 µg/mL, respectively. The TAT peptide alone exhibited no inhibitory activity against *S. flexneri*, as observed via the time-kill kinetics assay, despite its higher net positive charge of +8. These findings suggest that the antimicrobial results from the conjugation of the two peptides, TAT-Octominin II.

Other studies have reported enhanced antimicrobial activity by conjugating CPPs to conventional AMPs. TAT has been conjugated with several conventional AMPs, such as the marine peptides N_2_ isolated from the marine invertebrate lugworm (*Arenicola marina*), which showed faster cellular internalization in *Salmonella typhimurium*. However, the TAT-N_2_ conjugate required a higher concentration to inhibit *S. typhimurium* compared to N_2_ alone [21]. Trifluoroacetic and thioacetal lysine-modified peptides, such as KP13, KP15, and KP24, were reported to inhibit *Staphylococcus aureus*, *E. coli*, and *Pseudomonas aeruginosa* through transcription retardation [22]. TAT peptide has also been used to engineer conventional peptides to increase their specificity towards specific targets, such as in nuclear-targeted drug delivery [23]. Another well-characterized CPP and R9 have been conjugated with manganin, an AMP found in African clawed frogs, and M15. The resulting conjugated peptides exhibited higher antimicrobial activity, with lower MIC and MBC values and higher DNA binding affinity, against several Gram-negative and Gram-positive bacteria [24].

AMPs exert their antimicrobial effects through diverse mechanisms, to inhibit microorganisms through direct killing or by activating the host’s immune responses. Direct killing involves damaging the cell membrane, targeting intracellular components including DNA, RNA, and mitochondria, disrupting membrane-associated processes, and binding to membrane receptors [25,26]. Membrane disruption in bacterial cells is initiated by the formation of hydrogen bonds between AMP residues, such as arginine and lysine, and the phosphate groups of lipids. Hydrophobic residues of AMPs can interact and penetrate the hydrophobic region of the cell membrane, leading to membrane thinning [27]. Although CPPs can facilitate cellular uptake of cargo without damaging the cell membrane. We observed that TAT-Octominin II disrupts the cell membrane of *S. flexneri*. The high content of arginine and lysine residues create strong electrostatic lipid bonds, disrupting cell membranes. In addition, conjugation of Octominin II altered the physicochemical properties and structural characteristics of TAT, which may also contribute to the antimicrobial activity of TAT-Octominin II through membrane disruption.

The bacterial cell membrane protects bacterial cells by acting as a barrier that restricts and regulates the entry of external agents. On the other hand, a sufficient concentration of antibiotics must penetrate cell membrane of microorganism to exert its antimicrobial action [28,29]. Many multidrug-resistant bacteria have mechanisms that alter membrane permeability, thereby preventing or reducing the permeability of antibiotics. This leads to the requirement of higher antibiotic concentrations [30]. Therefore, studies have been conducted to increase membrane permeability to increase the delivery and effectiveness of antibiotics while overcoming antibiotic resistance [31,32]. Moreover, increased cell permeability can lead to the leakage of intracellular constituents, including DNA, RNA, and ions such as Ca^2+^ and Mg^2+^, thereby leading to cell death. Furthermore, changes in cell membrane permeability that cannot be adequately regulated by the microorganism can induce physiological stress within the cells, ultimately leading to cell death [33].

In our study, we observed that TAT-Octominin II increased bacterial cell membrane permeability, as demonstrated by the ONPG assay, which indicated increased membrane permeability. We also observed that TAT-Octominin II treatment caused the leakage of intracellular constituents including nucleic acids. Moreover, the PI/FDA assay demonstrated that TAT-Octominin II altered cell membrane integrity of *S. flexneri*. Collectively, these findings suggest that TAT-Octominin II exerts its inhibitory activity by inducing physiological stress through ROS generation. ROS generation can affect cellular processes such as metabolism and protein synthesis. It also directly damages DNA and RNA and impair transcription process of bacteria cells [34]. Magnus et al. (2019) investigated the effect of Cathelicidin peptides on several Gram-negative and Gram-positive microorganisms such as *E. coli*, *P. aeruginosa*, *S. aureus*, and *Enterococcus faecalis* and concluded that Cathelicidin exerts its antimicrobial activity by restricting cell growth through membrane perturbation and ROS induction [35].

Gene expression analysis following treatment with TAT-Octominin II against *S. flexneri*, focusing on the expression pattern of antioxidant genes, such as sodA, sodB, katG, oxyR, and soxR, revealed that TAT- Octominin II treatment upregulated expression of antioxidant genes. This result indicates that *S. flexneri* cells attempted to reduce the oxidative stress that progressed inside the cells after the TAT-Octominin II treatment. The genes soxR and oxyR are activated when their sensor proteins are directly oxidized, and these regulators the expression of other genes that mediate antioxidant defense, such as superoxide dismutase, catalases, and thioredoxins [36]. We observed that the 5 µg/mL (0.5 MIC) treatment showed greater upregulation of the tested genes than 10 µg/mL (MIC). This can occur when cells perform repair mechanisms by upregulating relevant genes at low levels. However, at a lethal concentration of the peptide have several mechanisms to cause strong inhibitory effects, which may attenuate or dysregulate gene expression responses. In addition, TAT-Octominin II possesses nucleotide-binding activity that can cause defects in the transcription process in the cell. Many AMPs were identified with DNA and RNA-binding ability that plays an important role in their inhibitory activity such as lactoferrin and histatin 5 against *Streptococcus mutans* [37]. NK- 18 derived from mammalian NK-Lysin, exhibits AMPs activity against *E. coli* [37], while bovine-derived AMPs indolicidin also exhibits antimicrobial activity [38].

LPS are major components of the outer membrane of the cell envelope of Gram-negative bacteria. LPS are responsible for the host endotoxic reactions, which activate inflammatory pathways by inducing cytokine and septic shock [39]. LPS are highly rigid and less permeable than phospholipids. Therefore, LPS neutralization reduces the pathogenicity of microorganisms. Therapeutic strategies focus on neutralizing LPS molecules to treat sepsis and septic shock caused by Gram-negative bacteria [40]. Several AMPs, such as magainin analogs, pardaxin, folliculin, and Cathelicidin LL-37, have been reported to process LPS-neutralizing activity and to reduce LPS-mediated inflammation [41]. TAT-Octominin II also exhibited a concentration-dependent neutralizing activity. This may be due to the high cationic nature and hydrophobicity of the peptide, where a high net positive charge facilitates the formation of strong electrostatic interactions and neutralizes its negative charge, and hydrophobicity may promote peptide insertion into the LPS [41,42]. However, the exact mechanism by which TAT-Octominin II neutralizes LPS requires further investigation, since the physical conformation of the peptide may change depending on the physicochemical properties of the surrounding media.

TAT-Octominin II displayed increased cytotoxicity against Raw 264.7 cells and HEK293 cells, while showing low hemolytic activity. The similar characteristics have been reported for model amphipathic peptide (MAP) and transportan 10 peptides, which showed higher in vitro cytotoxicity and lower hemolytic activity [43]. However, the effective concentrations of TAT-Octominin II showed relatively low cytotoxicity. This observed cytotoxicity may be due to the high cationic charge of the peptide. A high positive net charge of the peptides can destabilize the cell membranes consequently induce cellular damage. In addition peptide aggregation may also contribute to increased cytotoxicity [44]. Hence, further studies are needed to understand the physicochemical characteristics and reactions of TAT-Octominin in different physicochemical environments.

Our attempt to enhance the antimicrobial activity of Octominin II through conjugation with a CPP was successful, as TAT-Octominin II exhibited enhanced antibacterial activity at a relatively low dose. The cumulative effects of increased membrane permeability and membrane damage may lead to increase oxidative stress in *S. flexneri*, thereby contributing to the antibacterial activity of TAT-Octominin II. Moreover, TAT-Octominin II exhibited DNA and RNA-binding activity, which may interfere with transcriptional regulation. In addition, TAT-Octominin II exhibited LPS-neutralizing activity, which may help mitigate endotoxin-mediated inflammatory effects associated with Gram-negative bacteria, including *S. flexneri*. Because TAT-Octominin II appears to act through multiple mechanisms, including membrane disruption, oxidative stress induction, nucleic acid interaction, and LPS neutralization, its antibacterial activity may not depend on a single bacterial target. Such multitarget activity may reduce the likelihood of rapid resistance development, as bacterial adaptation would require coordinated changes in several cellular pathways. A limitation of the present study is the absence of unconjugated Octominin II and TAT-alone controls in the membrane-permeability and nucleic-acid leakage assays; therefore, the individual contribution of each component to these effects could not be directly distinguished. However, this possibility should be confirmed in future long-term resistance-evolution studies. Further studies are also needed to evaluate and minimize toxicity-associated effects by systematically investigating the physicochemical factors that influence peptide toxicity.

## 4. Materials and Methods

### 4.1. Bacteria Strain Identification and Culture Conditions

The *S. flexneri* isolate was accurately sequenced using 16s rRNA sequencing with 27F (5′-AGAGTTTGATCATGGCTCAG-3′) and 1497R (5′-GGTTACCTTGTTACGACTT-3′) forward and reverse primers. The sequencing results were compared with those in the EzBioCloud database [45]. Moreover, a phenotypic test (motility assay) was performed to reconfirm the bacteria. Luria-Bertani (LB) culture plates were prepared by adding LB agar (bottom layer) and LB soft agar (4% agar) to the plates. One drop of the suspected Shigella culture and a previously confirmed *E. coli* culture (OD = 0.07 at 595 nm) were placed in the middle of two separate plates and incubated at 37 °C for 24 h, and the motility patterns were observed [46]. The confirmed *S. flexneri* strain was stored in an LB medium with 40% glycerol (*v*/*v*) at −80 °C until use.

### 4.2. Designing and Synthesis of TAT-Octominin II

TAT-Octominin II was designed by conjugating the HIV-TAT peptide (YGRKKRRQRRR) to the N-terminus of the original Octominin II peptide (GWLIRGAIHAGKAIHGLI). The three-dimensional structure was determined using the PEPstrMOD online tool and visualized using the Biovia Discovery Studio visualizer (Biovia, Dassault Systems, Vélizy-Villacoublay, France: version 2021). The physicochemical properties of the peptides were determined using the peptide R package [47]. TAT-Octominin II was synthesized using solid-phase peptide synthesis (AnyGen, Gwangju, Republic of Korea). It was purified using reverse-phase high-performance liquid chromatography. The peptide was dissolved in nuclease-free water (1 mg/mL) and stored at −20 °C until further use.

### 4.3. Determination of Minimum Inhibitory Concentration (MIC) and Minimum Bactericidal Concentration (MBC)

An overnight culture of *S. flexneri* was prepared in sterile LB medium, after which a 10% inoculum was transferred into fresh LB broth to obtain a bacterial density of approximately 1 × 10^6^. CFU/mL. Aliquots of the bacterial suspension (2 mL) were exposed to TAT-Octominin II at concentrations of 5, 10, and 15 µg/mL and incubated at 37 °C with shaking at 180 rpm for 18 h. Gentamicin (25 µg/mL) was included as the positive control. Following incubation, bacterial growth was assessed visually based on culture turbidity, and the lowest peptide concentration that produced no visible turbidity was recorded as the minimum inhibitory concentration (MIC). For determination of the minimum bactericidal concentration (MBC), 100 µL aliquots from the treated cultures were spread onto LB agar plates and incubated at 37 °C for 24 h. The lowest concentration that resulted in no visible colony growth on the agar plates was defined as the MBC.

### 4.4. Time-Kill Kinetics, pH-Dependent Activity, and S. flexneri Survival upon TAT-Octominin II Treatment

A fresh *S. flexneri* suspension was prepared by transferring 10% of an overnight culture into sterile LB medium and adjusting the bacterial density to approximately 1 × 10^6^ CFU/mL. The suspension was dispensed into a 96-well plate and exposed to TAT-Octominin II at final concentrations of 5, 10, 15 and 25 µg/mL in a total volume of 200 µL. Cultures were incubated at 37 °C for 18 h, and bacterial growth was monitored at 3 h intervals by recording the OD at 595 nm using an iMark^TM^ microplate reader (Bio-Rad, Hercules, CA, USA). Gentamicin (25 µg/mL) served as the positive control.

The influence of pH on the antibacterial activity of TAT-Octominin II was examined over a pH range of 3–8. *S. flexneri* suspensions adjusted to 1 × 10^6^ CFU/mL were treated with 0, 5, 10, 15, or 25 µg/mL TAT-Octominin II and incubated at 37 °C for 18 h under the respective pH conditions. Bacterial growth was subsequently quantified by measuring absorbance with an iMark^TM^ microplate reader (Bio-Rad, Hercules, CA, USA). The percentage of growth inhibition at each pH was calculated relative to the corresponding untreated control, which was considered to represent 0% inhibition.

Bacterial viability following TAT-Octominin II treatment was further evaluated using an MTT assay. *S. flexneri* cultures at 1 × 10^6^ CFU/mL were exposed to 5, 10, or 15 µg/mL TAT-Octominin II for 24 h at 37 °C. Following treatment, the cells were harvested by centrifugation at 1500× *g* for 10 min and washed with 1× phosphate-buffered saline (PBS). The resulting cell pellets were incubated with 20 µL of MTT solution (5 mg/mL; Sigma-Aldrich, St. Louis, MO, USA) at 37 °C for 30 min. Subsequently, 50 µL of dimethyl sulfoxide was added to each sample and thoroughly mixed. Absorbance was then recorded at 595 nm using an iMark^TM^ microplate reader (Bio-Rad, Hercules, CA, USA).

### 4.5. Determination of Morphological Changes by TAT-Octominin II via FE-SEM

Morphological changes in *S. flexneri* following TAT-Octominin II treatment were examined using field-emission scanning electron microscopy (FE-SEM; SU7000, Hitachi, Tokyo, Japan). Fresh *S. flexneri* cultures adjusted to 1 × 10^6^ CFU/mL were exposed to TAT-Octominin II at 0 µg/mL, the MIC (10 µg/mL), or the MBC (25 µg/mL). Gentamicin (25 µg/mL) was included as the positive control. After incubation at 37 °C for 10 h, the bacterial cells were harvested by centrifugation at 1500× *g* for 10 min. The cell pellets were then washed twice with 1× PBS and dehydrated sequentially in 30%, 50%, 70%, 80%, 90%, and 100% ethanol. Following dehydration, the samples were coated with platinum by ion sputtering and examined using the SU7000 FE-SEM system.

### 4.6. Determination of Inner Cell Membrane Permeability of TAT-Octominin II by ONPG Assay

The inner membrane permeability of TAT-Octominin II was determined by measuring the level of O-nitrophenol produced by cleaving ONPG by the β-galactosidase enzyme released by *S. flexneri*. The assay was performed according to the method described by Gravel et al. (2017) [48]. Briefly, *S. flexneri* culture (1 × 10^6^ CFU/mL) cells were treated with TAT-Octominin II (0, 0.25 MIC; 2.5 µg/mL, 0.5 MIC; 5 µg/mL, and MIC; 10 µg/mL) and 0.5 mg/mL of ONPG was added for each sample. The bacterial cultures were then incubated at 37 °C for 3 h at 180 rpm. Every 15 min, 1 mL of the sample was collected and centrifuged at 5000× *g* for 1 min, and the supernatant was collected for analysis. The absorbance was determined at 420 nm using a Biorad iMarkTM microplate reader at 15 min intervals for 3 h (Bio-rad, Hercules, CA, USA).

### 4.7. Determination of Nucleotide Leakage upon TAT-Octominin II Treatment

Nucleotide leakage assay was performed as described by Zhang et al. (2022) [49]. A fresh *S. flexneri* suspension was prepared in LB broth at a final density of 1 × 10^8^ CFU/mL. The bacterial cultures were exposed to TAT-Octominin II at the MIC (10 µg/mL) and MBC (25 µg/mL) and incubated at 37 °C for 1 h. During the incubation period, 1 mL aliquots were withdrawn at 15 min intervals and centrifuged at 6000× *g* for 1 min. The resulting supernatants were collected, and nucleic acid leakage was evaluated by measuring absorbance at 260 nm using an Optizen^TM^ POP spectrophotometer (K Lab Co., Daejeon, Republic of Korea).

### 4.8. Determination of Membrane Permeability/Integrity Changes by PI/FDA Assay

A fresh *S. flexneri* culture adjusted to 1 × 10^6^ CFU/mL was exposed to TAT-Octominin II at 0 µg/mL, the MIC (10 µg/mL), or the MBC (25 µg/mL), while gentamicin (25 µg/mL) served as the positive control. Following incubation at 37 °C for 12 h, bacterial cells were harvested by centrifugation at 5000× *g* for 10 min at 4 °C and resuspended in 1 mL of 1× PBS. For PI/FDA staining, the bacterial suspensions were supplemented with 8 µL of FDA (5 mg/mL, Sigma Aldrich, Munich, Germany) and 50 µL of PI (2 mg/mL; Sigma Aldrich, Munich, Germany), followed by incubation in the dark for 30 min. The stained cells were subsequently collected by centrifugation at 5000× *g* at 4 °C and washed with PBS to remove unbound dye. The resulting pellets were resuspended in 10–20 µL of PBS, and 5 µL of each suspension was mounted on a glass slide, covered with a coverslip, and sealed. Fluorescence images were acquired using an LSM 880 confocal microscope with Airyscan (Carl Zeiss, Jena, Germany). PI fluorescence was detected using excitation and emission wavelengths of 535 and 617 nm, respectively, whereas FDA fluorescence was recorded at excitation and emission wavelengths of 488 and 535 nm, respectively.

### 4.9. Determination of Reactive Oxygen Species Generation by TAT-Octominin II

Intracellular ROS production was assessed using 2′,7′-dichlorofluorescein diacetate (H_2_DCFDA). Fresh *S. flexneri* cultures adjusted to 1 × 10^6^ CFU/mL were exposed to TAT-Octominin II at 0 µg/mL, the MIC (10 µg/mL), or the MBC (25 µg/mL), while gentamicin (25 µg/mL) served as the positive control. After incubation at 37 °C for 12 h, bacterial cells were harvested by centrifugation at 5000× *g* for 10 min at 4 °C. The pellets were washed with 1× PBS and resuspended in 1 mL of 1× PBS. Subsequently, 60 µL of H_2_DCFDA solution (1 mg/mL) was added to each cell suspension, mixed thoroughly, and incubated in the dark for 30 min. The stained cells were then collected by centrifugation at 5000× *g* for 10 min at 4 °C and washed with 1× PBS to remove excess dye. Finally, the cells were resuspended in 1× PBS, and 50 µL of each suspension was mounted on a glass slide and covered with coverslip. Fluorescence was visualized using confocal laser scanning microscopy, with excitation and emission wavelengths of 488 and 535 nm, respectively.

### 4.10. Determination of the Antioxidant Gene Expression upon TAT-Octominin II

For mRNA analysis, *S. flexneri* (1 × 10^6^ CFU/mL) was treated with 0, 5, and 10 µg/mL of TAT-Octominin-II and then incubated at 37 °C for 3 h. RNA was isolated using an Accuprep^®^ bacterial RNA extraction kit, according to the manufacturer’s protocol (Bioneer, Daejeon, Republic of Korea). RNA concentrations were measured using NanoDrop (Thermo Fisher Scientific, Waltham, MA, USA), and cDNA was prepared using the TAKARA PrimeScript 1st Strand cDNA Synthesis Kit (TaKaRa, Katsu, Shiga, Japan). q-RT-PCR was performed using the designed primers (Supplementary Table S1) for selected genes using the Thermal Cycler Dice Real Time System III (TaKaRa, Katsu, Shiga, Japan). A three-step PCR reaction was performed with a single dissociation step. The relative mRNA expression fold values were calculated using the 2^−(ΔΔCT)^ method [50].

### 4.11. Determination of the Nucleic Acid Binding Ability of TAT-Octominin II

Genomic DNA was extracted from *S. flexneri* (2 × 10^8^ CFU/mL) using the DNA extraction kit according to the manufacturer’s instructions (Bioneer, Daejeon, Republic of Korea). DNA purity and concentration were analyzed using a NanoDrop microvolume spectrophotometer by determining the DNA concentration and OD260/OD280 (Thermo Fisher Scientific, Waltham, MA, USA). DNA gel retardation assay was performed according to the method described by Hellman et al. (2007) [51]. Briefly, 2 µg of genomic DNA was mixed with TAT-Octominin II at different weight ratios (1:0,1:0.5, 1:1, and 1:2 *w*/*w*). The reaction mixture was then incubated at 37 °C for 30 min. For the RNA-binding assay, 2 µg of RNA was reacted with different ratios of TAT-Octominin II (1:0, 1:0.25,1:0.5, and 1:1 *w*/*w*). The reaction was incubated at 37 °C for 30 min. After incubation of both samples, gel electrophoresis was performed using the Mupid^®^—2 plus electrophoresis system (TAKARA, Shiga, Japan), and the gel image was visualized using the MaXidoc Gel imaging system (Daihan Scientific, Daegu, Republic of Korea).

### 4.12. LPS Neutralization Assay

LPS neutralization assay was performed according to the method described by Jayathilaka et al. 2021 [52] using the Pierce^TM^ LAL Chromogenic Endotoxin Quantitation Kit (Thermo Fisher Scientific, Waltham, MA, USA). For the LPS-neutralization assay, a 0.5 ng/mL LPS standard solution was prepared from the *E. coli* endotoxin standard using the endotoxin-free water supplied with the LAL chromogenic assay kit. Aliquots of the LPS solution (50 µL) were mixed with an equal volume (50 µL) of TAT-Octominin II at concentrations ranging from 0 to 100 µg/mL. The reaction mixtures were incubated at 37 °C for 30 min. Following incubation, the residual LPS level was quantified according to the manufacturer’s instructions for the LAL chromogenic assay. The percentage of LPS neutralization was subsequently calculated using the following equation:

LPS neutralization % = [1 − (Ab test/Ab negative control)] × 100%

Ab test represents the absorbance value of the TAT-Octominin II-treated sample, and Ab negative control represents the absorbance values of the endotoxin-free water -treated sample.

### 4.13. In Vitro and In Vivo Toxicity, and Hemolysis Activity of TAT-Octominin II

In vitro cytotoxicity was measured using Raw 264.7 and HEK-293 cells. Cells were cultured in Dulbecco’s modified Eagle’s medium (Welgene, Gyeongsangbuk-do, Republic of Korea) containing 10% (*v*/*v*) fetal bovine serum (Welgene) with an antibiotic-antimycotic solution (Thermo Fisher Scientific, Waltham, MA, USA) and incubated at 37 °C in a humidified atmosphere of 5% CO_2_ for 24 h. Subsequently, the cells were seeded into a TC 96-well plate at a density of 1 × 10^4^ cells per well (100 µL) and allowed to adhere by incubation at 37 °C for 24 h. The cell media was then aspirated, and the cells were treated with TAT-Octominin II (0–100 µg/mL) with fresh media. The cells were then incubated at 37 °C for 24 h. Cell viability was measured using the EZ-Cytox cell viability assay kit (DoGenBio Co., Seoul, Republic of Korea) according to the manufacturer’s protocol. Untreated cells were considered negative controls. In vivo toxicity of TAT-Octominin II was assessed by treating zebrafish (*Danio rerio*) larvae (72 hpf). Zebrafish larvae (60 larvae) were randomly allocated to six experimental groups, including the PBS control and five TAT-Octominin II groups (1, 2, 5, 10, and 20 µg/mL), with 10 larvae assigned to each group (*n* = 10/group). The larvae were placed individually in the wells of round-bottom 96-well plate. Then, TAT-Octominin II was added to the wells (0–20 µg/mL). Subsequently, the larvae were maintained at 28 °C and observed mortality for 96 h. No animals, experimental units, or data points were excluded from any experimental group or analysis. Moreover, potential confounding factors related to treatment order, measurement order, and well location were not specifically controlled. The hemolytic activity of TAT-Octominin II was determined using murine red blood cells (RBCs) according to the method described by Jayathilaka et al. (2021) [52]. Briefly, the collected RBCs were washed with 1× PBS and treated with different concentrations of TAT-Octominin II (0–100 µg/mL). One percent of Triton X-100 (Sigma Aldrich, Munich, Germany) was used as a positive control. Treated RBCs were incubated at room temperature for 1 h, and the supernatant was separated by centrifugation at 5000× *g*. The absorbance of the supernatant was measured at 415 nm using a Biorad iMarkTM microplate reader (Biorad, Hercules, CA, USA). Hemolysis activity was calculated using the following equations,

AB Triton = absorbance of the Triton X-100 treated sample,

Hemolysis % = [(Ab test − Ab PBS)/(Ab triton − Ab PBS)] × 100%

where Ab test = absorbance of the treated sample, and Ab PBS = absorbance of the PBS-treated sample.

### 4.14. Statistical Analysis

Statistical analysis of all experiments was conducted using GraphPad Prism software version 8.1 (GraphPad Software Inc., La Jolla, CA, USA). Each in vitro and in vivo assay was performed in triplicate and analyzed using a one-way ANOVA test. In both cases, statistical significance was determined at *p*-values less than 0.05 (*p* < 0.05) and 0.01 (*p* < 0.01), respectively.

## Figures and Tables

**Table 1:** Antibiotic resistance profile of *S. flexneri*.

Antibiotic Class	Antibiotic Name (Amount/Disk)	Antibiotic Sensitivity Level (Disc Diameter in mm)	Zone Diameter Interpretation Standard (mm) Resistant Intermediate Susceptible		
Aminoglycoside	Streptomycin (300 µg)	Resistant	≥6	7	≥10
	Gentamicin (120 µg)	Susceptible (19.6 ± 0.10)	≤6	7	≥10
	Amikacin (30 µg)	Resistant	≤14	15–18	≥17
	Clindamycin (2 µg)	Resistant	≤14	15–20	≥21
	Kanamycin (10 µg)	Resistant	≤13	14–18	≥18
	Tobramycin (10 µg)	Resistant	≤12	13–14	≥19
Tetracyclines	Tetracycline (30 µg)	Resistant	≤14	15–18	≥19
	Doxycycline (30 µg)	Resistant	≤13	12–15	≥16
Fluoroquinolone	Ciprofloxacin (5 µg)	Intermediate (16.9 ± 0.02)	≤14	15–18	≥19
	Ofloxacin (5 µg)	Intermediate (12.9 ± 0.27)	≤13	12–15	≥16
Glycopeptide	Vancomycin (30 µg)	Resistant	≤14	15–18	≥19
Macrolides	Erythromycin (15 µg)	Resistant	≤13	14–22	≥23
Cephalosporin	Cefotaxime (30 µg)	Susceptible (21.6 ± 0.10)	14	15–22	≥23
Penicillin	Penicillin (10 µg)	Resistant	14		≥15
	Ampicillin (10 µg)	Resistant	≤13	14–17	≥18
Carbapenem	Imipenem (10 µg)	Susceptible (20.8 ± 0.5)	≤13	14–17	≥18
Folic acid synthesis inhibitor	Sulfamethoxazole (23.75 µg)	Resistant	≤10	11–15	≥16
Chloramphenicol	Chloramphenicol (30 µg)	Resistant	≤12	13–17	≥18

**Table 2:** Physiochemical properties of Octominin II and TAT-Octominin II.

Property	Octominin II	TAT-Octominin II
Molecular weight (Da)	1833.5	3425.10
Net charge	+2.46	+10.00
Isoelectric point	11.66	12.40
Aliphatic index	134.38	91.93
Instability index	12.51	67.84
Boman index (kcal/mol)	−0.28	1.23
Hydrophobicity index	0.46	0.33

## Data Availability

The data presented in this study is available on request from the corresponding authors due to our future grant applications using the available data.

## Supplementary Materials

The following supporting information can be downloaded at https://www.mdpi.com/article/10.3390/antibiotics15101033/s1. Supplementary Figure S1. Differentiation between *E. coli* and *S. flexneri* based on a motility assay. Supplementary Figure S2. Viability assessment of *S. flexneri* following treatment with TAT-Octominin II. Supplementary Table S1: Primers used in this study.
