# Supplementary material for: Cell-Penetration Peptide-Conjugated Octominin II Hybrid Inhibits the Multidrug-Resistant Shigella flexneri
Source: Antibiotics (Basel). 2026 Oct 16;15(10):1033. doi: 10.3390/antibiotics15101033 (PMC13700547; doi:10.3390/antibiotics15101033)
Supplement: Supplementary file 1 [file antibiotics-15-01033-s001.zip › antibiotics-4532413-supplementary.pdf]

Supplementary Materials

Supplementary Table S1: Primers used in this study

Gene name	Forward primer (5′-3′)	Reverse primer	Accession number
***Shigella flexneri* 16S ribosomal RNA**	CCCCCTGGACGAAGACTG	GTGGACTACCAGGGTATCTAATCCT	AE005674.2
**Catalase/hydroperoxides HPI(I) (katG)**	GTGGTCATACGCTGGGTAAA	CCCAGCCTAAACCTTGTTCT	AAN45453.2
**Superoxide dismutase A (sodA)**	CGCTGATGGGTGAAGCTATT	ACGGCGGTTCTGGAATTT	AE005674.2
**Superoxide dismutase B (sodB)**	ACTACGGCAAGCATCATCAG	CACCTTCAGAGCTGCGAATAA	AE005674.2
**DNA-binding transcriptional regulator (oxyR)**	CAGGTAGCGGGATCACTTTATT	GACGATAAACCAGGCCAATAGT	AE005674.2
**Redox-sensitive transcriptional activator (soxR)**	GGCATACGTTAAGTGCGAAAG	ACCAATACATCCGTCCAGTTC	AE005674.2

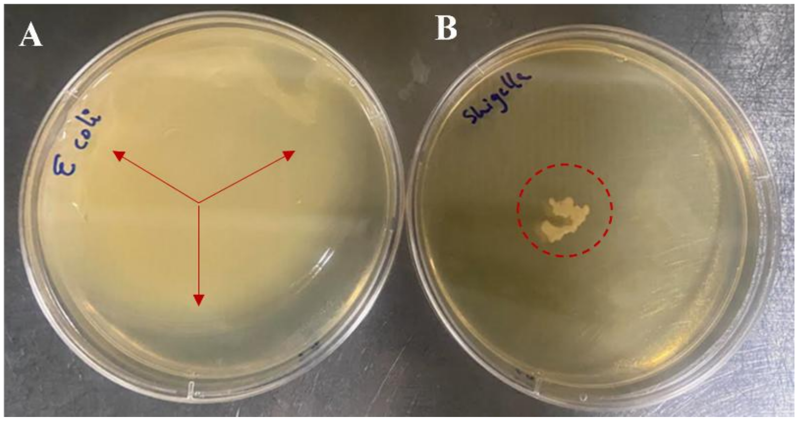

Supplementary Figure S1. Differentiation between *E. coli* and *S. flexneri* based on a motility assay. Culture plates were prepared with an LB agar layer (bottom) and a soft agar layer (4% agar). A drop of the previously identified *E. coli* culture and the suspected *Shigella* sp. culture (OD_{595}=0.07) was placed in the center of each plate and incubated at 37 °C for 24 h. The motility patterns were then observed. A; *E. coli*: motile showing growth spreading throughout the culture plate; B; *Shigella* sp.; non-motile, showing grown only at the center of the culture plate.

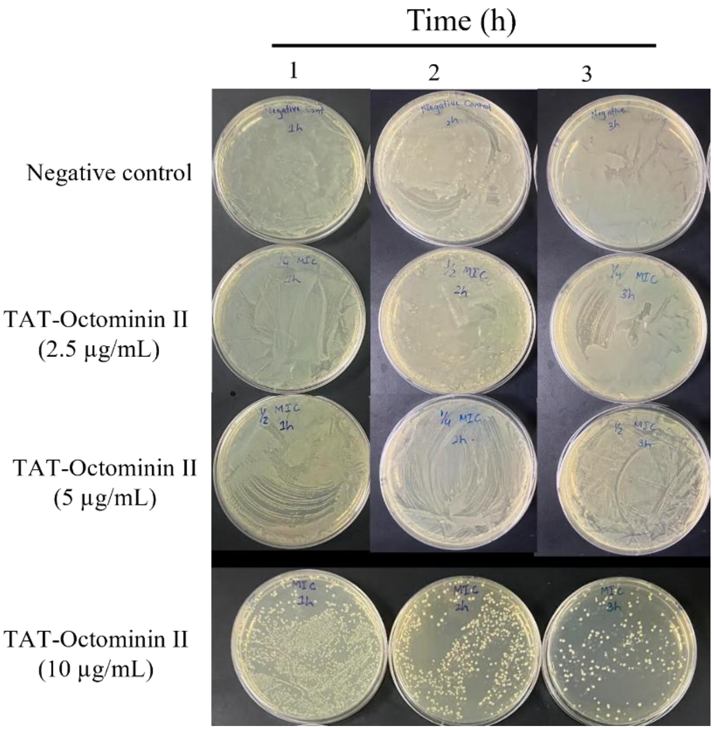

Supplementary Figure S2. Viability assessment of *S. flexneri* following treatment with TAT-Octominin II. The viability of *S. flexneri* was assessed during the cell permeability assay using the ONPG. *S. flexneri* (100 µL) was treated with TAT-Octominin II at 0, 2.5, 5, and 10 µg/mL. After 1 h post treatment, the cultures were incubated for additional treatment 3 h. Next 100 µL of each culture was plated onto LB agar and incubated at 37 °C for 24 h. Then the growth of *S. flexneri* was assessed to determine whether increased cell permeability was associated with *S. flexneri* cell death.
